# Kaposi's Sarcoma Presenting as Acute Small Bowel Obstruction Diagnosed on Multidetector Computed Tomography with Histopathological Correlation

**DOI:** 10.1155/2015/581470

**Published:** 2015-03-17

**Authors:** Jaydeep Halankar, Elaine Martinovic, Paul Hamilton

**Affiliations:** Department of Medical Imaging, Sunnybrook Health Sciences Centre (Affiliated to University of Toronto), 2075 Bayview Avenue, Toronto, ON, Canada M4N 3M5

## Abstract

Kaposi's sarcoma was originally described by Moritz Kaposi in 1872 as a rare form of multiple hemorrhagic skin lesions. Today it is well documented as a systemic, multifocal, steadily progressive reticuloendothelial system tumor with a predilection for skin and visceral involvement. It occasionally presents as a visceral disease without skin manifestations. We report a case of Kaposi's sarcoma of the small bowel in a seropositive patient who presented with acute right lower quadrant pain and was diagnosed with intestinal obstruction with perforation on contrast-enhanced multidetector computed tomography (MDCT). The diagnosis was confirmed as Kaposi's sarcoma on postoperative histopathological analysis.

## 1. Case Report

Over one hundred years ago Moritz Kaposi described an unusual hemorrhagic cutaneous lesion, which has been known since as Kaposi's sarcoma [1]. Visceral involvement which occurs in a small number of patients (less than 10 percent) with skin lesions is considered as a multicentric focal manifestation of the same disease rather than metastasis from the skin. Kaposi's sarcoma is considered today as a systemic multifocal steadily progressive tumor of the reticuloendothelial system. It has been reported to affect almost every organ in the body and occasionally presents as a visceral disease without skin manifestations [2]. We report a case of acute small bowel obstruction with perforation from Kaposi's sarcoma diagnosed on MDCT with histopathological correlation.

A 59-year-old man infected with the human immunodeficiency virus (HIV) presented to the emergency department with severe abdominal pain localized to the right lower quadrant of approximately 12-hour duration. This was accompanied by two episodes of vomiting and decreased appetite. The patient was also getting treatment for pulmonary tuberculosis.

Clinical examination showed pain in the right lower quadrant. He was found to be febrile with a temperature of 38.1°C (100.6°F) and laboratory findings showed a normal white blood cell (WBC) count. A MDCT was performed using oral and intravenous contrast.

**Figure 1:**
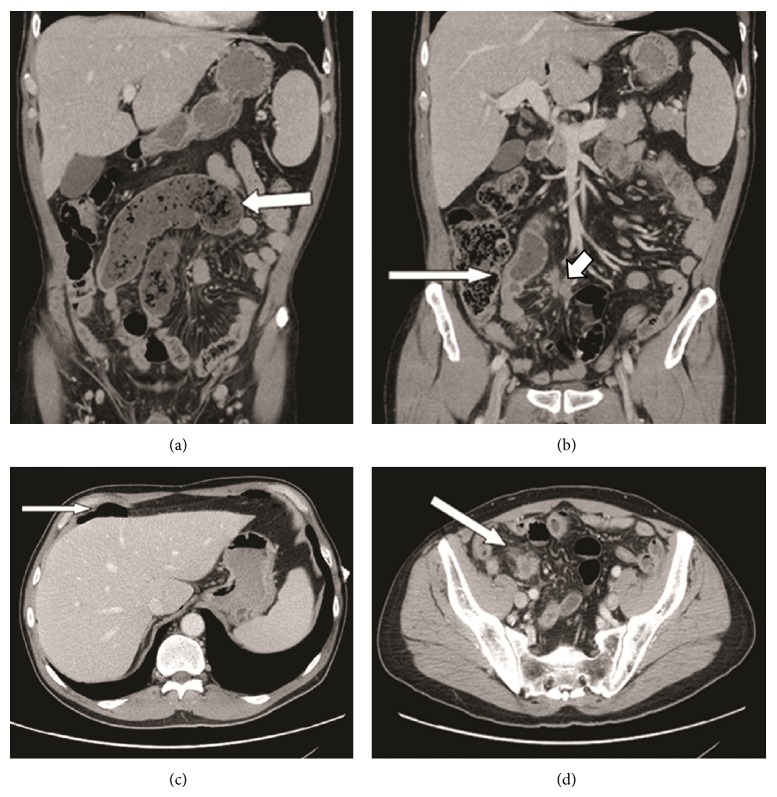
(a) Coronal contrast-enhanced CT image shows a markedly dilated fluid filled small bowel loop in the mid to right abdomen (*arrow*). There is evidence of stasis with small bowel fecalization sign. (b) Coronal contrast-enhanced CT image shows point of transition with associated irregularity and thickening of the bowel wall (*large arrow*). Note the abnormal enhancing lymph node (*small arrow*) in the adjacent small bowel mesentery (medially). (c) Axial contrast-enhanced CT image shows free intraperitoneal air below the ventral abdominal wall (*arrow*) in keeping with perforation. (d) Axial contrast-enhanced CT image shows a necrotic enhancing lymph node in the right lower quadrant (*arrow*).

The MDCT showed an abnormally dilated small bowel loop (up to 4.5 cm) in the right mid to lower abdomen with small bowel fecalization. There was an abrupt transition point in the right lower quadrant with wall irregularity and thickening at the level of the transition and a possible small intraluminal mass. There were also multiple locules of intraperitoneal free air, concerning for perforation; however no definite site was identified. Inferior to the transition point within the small bowel mesentery there were multiple enhancing soft tissue masses (largest measuring 2.6 × 2.2 cm) in keeping with abnormal lymphadenopathy (Figure 1).

A preoperative diagnosis of acute mechanical small bowel obstruction was made. The differential diagnoses under consideration were small bowel lymphoma, tuberculosis, fungal infection, small bowel carcinoid, and Kaposi's sarcoma. The imaging findings were conveyed to the referring clinician and surgeon.

An open surgical laparotomy showed proximal ileal small bowel microperforation along the antimesenteric border. Distal to the perforation there was an obstructing rubbery soft tissue intraluminal mass with associated varying size mesenteric lymphadenopathy. The diseased segment of small bowel was resected followed by a side to side anastomosis.

The intraoperative findings were concerning for small bowel lymphoma. The bowel specimen and lymph nodes were sent for histopathological diagnosis. The postoperative course was uneventful.

Pathologic analysis was performed at our institute on the resected 20.5 cm long segment of small bowel. The segment measured 4 cm in circumference distally and was dilated proximally to 6.5 cm in circumference. Four centimeters from the distal end was a stricture with the adjacent mucosa displaying congestion and effacement of the plicae semilunares. Superficial to this region, the bowel serosa displayed a 4 cm wide area of congestion. Finally, adjacent to the stricture was a well-demarcated, submucosal nodule measuring 0.4 cm in greatest dimension.

**Figure 2:**
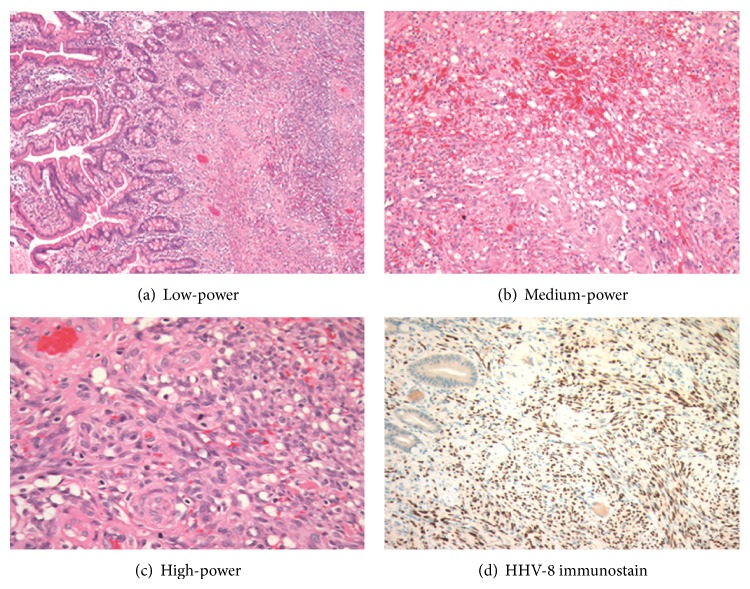
(a) Histologic section of small bowel (H&E). On the left, this low-power view shows normal intestinal villi protruding into the bowel lumen. On the right, beneath the villi, is a disorganized spindle-cell lesion. (b) Histologic section of small bowel (H&E). Extravasated blood is a prominent feature within the spindle-cell lesion, in this medium-power view. (c) Histologic section of small bowel (H&E). Small spaces, sometimes slit-like, are present within the spindle-cell proliferation. Mitotic activity is readily identified. (d) Histologic section of small bowel (HHV-8 immunostain). There is strong nuclear staining of the tumour cells.

Histology revealed this nodule to be a spindle-cell lesion involving the mucosa and submucosa and demonstrating characteristic slit-like spaces and extravasated red blood cells. Mitotic figures were easily identified. Immunohistochemical staining for HHV-8 demonstrated strong nuclear staining within the lesion (Figure 2). These features were diagnostic of Kaposi's sarcoma [3]. Evaluation of mesenteric lymph nodes demonstrated reactive plasma cell (Castleman-like) infiltrates and noncaseating granulomas.

A final diagnosis of Kaposi's sarcoma presenting as acute small bowel obstruction with perforation was made with histopathological correlation.

## 2. Discussion

Kaposi's sarcoma of the small bowel is an uncommon entity seen mostly affecting HIV positive patients. It has been described to occur mostly in young homosexual males and differ from its classical form in virulence and preponderance of systemic manifestations [4]. Most gastrointestinal manifestations of Kaposi's sarcoma have been documented using conventional barium procedures [4–6]. On the basis of our review of the literature, we believe this is an uncommon case of Kaposi's sarcoma of the small bowel diagnosed preoperatively as acute mechanical small bowel obstruction with perforation.

Kaposi's sarcoma is the most common AIDS related tumor in homosexual men and in populations in parts of Africa [6]. The gastrointestinal tract is the third most common site of Kaposi's sarcoma after the skin and lymph nodes [5, 6]. Kaposi's sarcoma is clinically silent and occurs concurrently with or after cutaneous disease; however, primary gastrointestinal Kaposi's sarcoma has been described [7].

The diagnosis of gastrointestinal Kaposi's sarcoma is made on endoscopic visualization of a submucosal mass or masses of a red-purple colour, which is typical of Kaposi's sarcoma; confirmation is made histologically with biopsy specimen [6].

On the basis of previous reports, the small bowel and stomach were common sites while the colon and particularly the esophagus were rarely affected. Very few cases of isolated gastrointestinal lesions without any other stigmata of systemic or skin manifestation have been reported [2].

The clinical presentation of Kaposi's sarcoma of the gastrointestinal system varies from constitutional symptoms to epigastric pain, generalized abdominal discomfort, and loss of weight and appetite [1, 4, 5]. Most lesions are found in asymptomatic individuals. More advanced lesions may present as gastrointestinal bleeding, protein losing enteropathy, diarrhea, and infrequently intussusception, intestinal obstruction, and perforation [4]. Our case report showed a patient presenting with acute right lower quadrant pain and a surgical abdomen with a differential diagnosis of small bowel obstruction or appendicitis.

The most common radiological finding in the gastrointestinal tract is of multiple submucosal masses with or without central ulceration (“target” or “bull's-eye” lesions). Plaques-like lesions or small nodules are less common radiological manifestations. Intussusception and bowel obstruction are an uncommon manifestation of Kaposi's sarcoma but do occur [6].

MDCT was beneficial in the diagnosis of the acute small bowel obstruction in our patient and differentiating from other causes of right lower quadrant pain such as appendicitis, cecal diverticulitis, omental infarct, infectious ileitis, Meckel's diverticulitis, and inflammatory bowel disease of the distal small bowel.

As shown in this case the multiplanar reformatted 2D images with coronal and sagittal reconstructions clearly depicted the dilated small bowel loop, right lower quadrant transition, and intraperitoneal free air.

Kaposi's sarcoma is a tumour of spindle cells and vascular clefts with extravasated red blood cells. The spindle cells of Kaposi's sarcoma are generally believed to originate from lymphatic or blood vessel endothelial cells, which would explain the multicentric nature of the tumour [6]. The HIV regulatory protein, transactivator target (TAT), which is important for viral replication, is hypothesized to cause proliferation of the tumor cells [7].

The specimen underwent gross, histologic, and immunohistochemical assessment, revealing a spindle-cell lesion involving the mucosa and submucosa with slit-like spaces and extravasated red blood cells. Mitotic figures were readily identified. The tumour cells showed strong nuclear positivity for HHV-8 by immunohistochemistry. The features were diagnostic of Kaposi's sarcoma.

## 3. Conclusion

In closing, this case report documents the preoperative imaging diagnosis of acute mechanical small bowel obstruction with perforation in an HIV positive male. The appearances on MDCT suggest an obstructing intraluminal mass as the cause and Kaposi's sarcoma was confirmed on postoperative histopathological analysis.
